# The Statistical Neuroanatomy of Frontal Networks in the Macaque

**DOI:** 10.1371/journal.pcbi.1000050

**Published:** 2008-04-04

**Authors:** Bruno B. Averbeck, Moonsang Seo

**Affiliations:** 1 Sobell Department of Motor Neuroscience and Movement Disorders, Institute of Neurology, University College London, London, United Kingdom; 2 MSc Programme in Clinical Neuroscience, Institute of Neurology, University College London, London, United Kingdom; University College London, United Kingdom

## Abstract

We were interested in gaining insight into the functional properties of frontal networks based upon their anatomical inputs. We took a neuroinformatics approach, carrying out maximum likelihood hierarchical cluster analysis on 25 frontal cortical areas based upon their anatomical connections, with 68 input areas representing exterosensory, chemosensory, motor, limbic, and other frontal inputs. The analysis revealed a set of statistically robust clusters. We used these clusters to divide the frontal areas into 5 groups, including ventral-lateral, ventral-medial, dorsal-medial, dorsal-lateral, and caudal-orbital groups. Each of these groups was defined by a unique set of inputs. This organization provides insight into the differential roles of each group of areas and suggests a gradient by which orbital and ventral-medial areas may be responsible for decision-making processes based on emotion and primary reinforcers, and lateral frontal areas are more involved in integrating affective and rational information into a common framework.

## Author Summary

The anatomical input to a cortical area defines, to a large extent, the functions that the area can perform. For example, if an area has no visual inputs, it cannot carry out computations on visual information. Therefore, understanding the inputs to a patch of cortex can provide fundamental insight into the function of the area. Anatomical tract tracing studies in macaque monkeys have defined much of the connectivity between areas of the macaque brain. We compiled the information on the anatomical inputs to 25 cytoarchitectonically defined frontal cortical areas. In its raw form, this connectivity is immensely complex, and the dominant inputs to each area cannot be clearly seen. To reduce the complexity, we carried out hierarchical cluster analysis on the areas based upon their inputs. We found a statistically robust organization of the areas. Identified clusters corresponded to anatomically contiguous groups, including orbital, ventral-medial, dorsal-medial, ventral-lateral, and dorsal-lateral areas. Using these groups, we were able to clearly define the dominant inputs to each cluster. We found that ventral-lateral, ventral-medial, dorsal-medial, dorsal-lateral, and caudal-orbital groups were defined by exterosensory, limbic, motor, exterosensory, and chemosensory inputs, respectively. These inputs likely drive the physiological responses found in each area.

## Introduction

The advent and application of modern anatomical tract tracing methods has led to extensive mapping of connections between architectonically defined areas of the macaque brain, resulting in the generation of a massive amount of information on connectivity. Given the complexity of the cortex, however, this information is often overwhelming, providing little insight in its complete detail [1]. This raises the question of whether or not there is any underlying structure in the connectivity that can be extracted by the appropriate neuroinformatics tools. Several groups have pursued this question, compiling a database of connectivity and examining the statistical organization of brain networks, focusing mostly on the organization of visual areas but also examining other areas of the cortex [2]–[7]. These analyses have identified patterns of global organization in sensory networks [4] as well as found relations between clusters generated with anatomical and physiological techniques [2].

In this study we focused on frontal networks, which are often associated with reward and decision making processes. A wealth of anatomical information is available about these networks, and insight into the functional role of particular brain areas or clusters of areas can be gained by understanding their dominant anatomical inputs [8]. Thus we examined the clustering of areas in the lateral, orbital and medial sectors of the frontal lobe, based upon their inputs. We applied statistical hierarchical clustering algorithms, based upon a branching Gaussian diffusion process, to characterize these clusters. These tools have seen a long development in the study of phylogenetic relationships between species based upon measures of continuous traits [9]–[13], but have not previously, to our knowledge, been applied to anatomy data. This approach allowed us to define a statistically significant hierarchically organized set of clusters. Based upon this hierarchical organization, we divided the frontal areas into 5 groups, and examined the dominant inputs to each group.

## Results

We began by accumulating a connectivity matrix for the frontal cortex, including inputs from as many cortical and limbic areas as possible. The frontal areas modeled included lateral frontal areas rostral to the arcuate sulcus, medial cortical areas from 23 forward, not including primary, lateral or medial premotor motor areas, and the entire orbital cortex and the adjacent insular cortex. The insular cortex was included due to its apparent functional role in decision making, likely mediated by its interoceptive [14] and chemosensory anatomical inputs.

**Figure 1:**
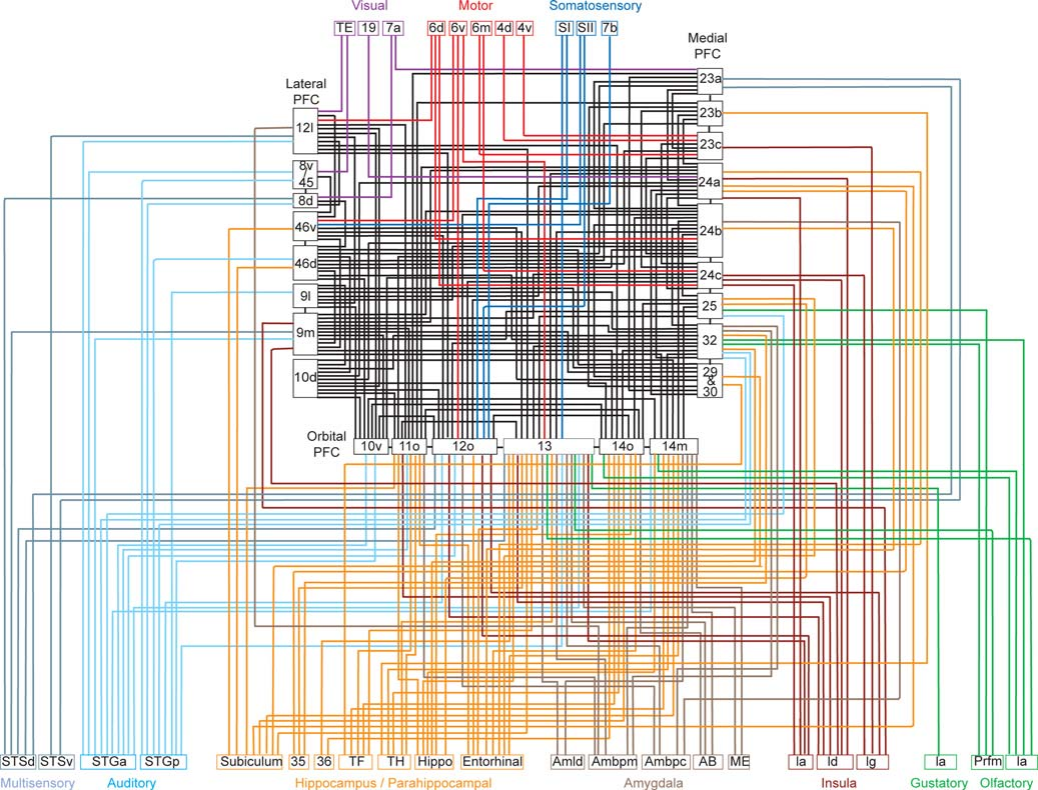
Connectivity diagram showing interconnections of frontal reward and decision-making networks with sensory, limbic, and motor systems. In this diagram, for clarity, only intermediate and strong projections to the frontal cortex are shown.

The frontal areas not only have dense interconnectivity, but they also receive information from every sensory modality including multisensory areas of the temporal lobe, as well as limbic and motor structures (Figure 1). The rich input and dense interconnectivity gives these areas the signals they need to carry out computation in support of decision making within any sensory modality, combining both interoceptive and limbic information, and the motor connections allow the expression of theses decision making tasks in their ultimate goal, action.

### Statistics of Interconnectivity

**Figure 2:**
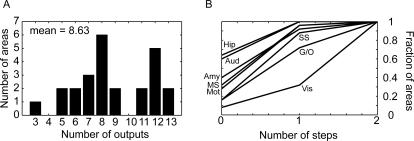
Connectivity of frontal areas. (A) Histogram showing count of areas with projections to the indicated number of areas. (B) Fraction of frontal areas that receive the signal from each modality as a function of the number of connectivity steps within frontal cortex. 0 indicates the areas which receive a direct projection from the indicated modality, and 1 indicates the fraction of areas that would receive the signal after a single step within frontal cortex. Mot, motor; Amy, amygdala; Hip, hippocampus; Vis, visual; SS, somatosensory; G/O, gustatory/olfactory; MS, multisensory; Aud, auditory.

We began by examining descriptive statistics of the connectivity. Although we mostly consider analyses based on inputs to each area, here we briefly analyze some statistics of the outputs of the areas. We found that, considering only the intermediate and strong connections, each frontal area sends outputs to an average of 8.6 other frontal areas. There was, however, a fairly broad distribution of numbers of outputs (Figure 2A). Although their appeared to be a bi-modal distribution of connectivity strengths, we did not find that it was related to any particular cluster of areas. Specifically, foreshadowing some of our later results (see below), we carried out an ANOVA analysis to see if clusters of areas tended to have more outputs. Thus, we considered clusters of dorsal medial (dmPFC), dorsal lateral (dlPFC), ventral medial (vmPFC), ventral lateral (vlPFC) and caudal orbital (coPFC) areas. Although we found that on average they tended to send more or less outputs to other frontal areas (9.0, 9.2, 8.9, 6.7 and 9.4 respectively), these differences were not statistically significant in a one-way ANOVA (p = 0.7503, main effect of cluster id, type III sum-of-squares to control for unequal data at each factor level). It is interesting to note, however, that areas 10d, 46v, 46d, 9m and 9l, which were spread across multiple clusters, but are closely positioned on the cortical surface, all were highly interconnected (i.e. 12 or 13 connections).

Given that each area connects to about 9 other areas with an intermediate or strong connection, the number of areas *n* to which a given input will flow is given by *n = 9^m^*, where *m* is the number of steps. Given this, input information from sensory, motor and limbic areas can reach everywhere within the frontal network (25 areas) within 2 steps (Figure 2B). Visual input appears to reach the fewest areas in a single step. This is due to the fact that the areas that receive intermediate and strong visual input (areas 12l and 45) both have relatively few reported connections. This analysis also predicts that there should be a temporal order to the flow of information within frontal areas. Specifically, areas that directly receive information from a specific modality, for example gustatory information in caudal orbital prefrontal cortex, should have responses to this type of stimulus before other areas.

We also found that connectivity within frontal cortex tended to be dominated by anatomically local interactions, where neighbors were defined as the spatial neighbors in our prefrontal map. Thus, each area was directly connected with 94% of its direct (first degree) neighbors, but only 57% of its neighbor's neighbors (not including its direct neighbors) and 32% and 19% of its third and fourth degree neighbors. (Incidentally, beyond 4 degrees there were no additional neighbors.)

### Clustering of Areas Based Upon Inputs

We next examined the hierarchical clustering of areas. This analysis clusters together areas that have similar inputs. A major goal of our analyses was to show that we can not only identify clusters, but that these clusters are statistically reliable, and reflect an underlying clustered organization of the connectivity. To achieve this we used a clustering algorithm which allowed us to measure how well each tree modeled the actual data. The metric of fit was the log-likelihood, which is similar to the R^2^, or more specifically the residual sum of squares, in regression. By comparing the distribution of this statistic for different trees, we could find the best tree and see if certain trees fit the data significantly better than other trees.

**Figure 3:**
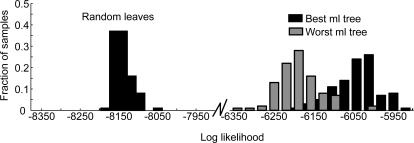
Log likelihood of trees. Distribution of log-likelihood values from 100 bootstrap datasets for most-likely tree, least most-likely (1001st) tree, and a random tree, generated by shuffling the leaves of the most likely tree.

**Figure 4:**
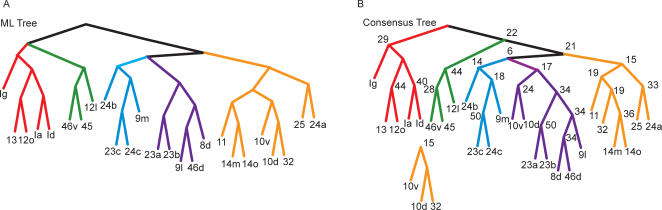
Trees fit to the data. (A) Most likely (ML) tree (highest likelihood), generated from boot-strap analysis. Colors indicate clusters into which we split the data for further analysis. (B) Consensus tree, generated from the 50 top most likely trees. Numbers at each branch point indicated how many times each cluster occurred in the 50 most likely trees. The detached cluster below the tree (10v,10d,32), which was part of ML tree, was not part of the consensus tree, although it occurred 15 times.

Although there are techniques for generating plausible trees, there is no direct way to guarantee that one has the single best tree for a particular dataset. Thus, we generated a set of 1001 candidate trees (see Methods) and assessed their fit to the data. We sorted the trees based upon their fit (the log-likelihood), and examined the fit of the best and worst of our candidate trees (Figure 3). The distribution of the likelihood for the best and worst trees that we identified showed overlap (Figure 3), but the best tree (henceforth the ML tree) was clearly superior to the worst tree in our candidate set. Next, we wanted to see whether or not the ML tree captured a statistically significant portion of the variance in our data. To do this, we compared the ML tree to a random tree generated by scrambling the leaves on the ML tree (Figure 3; Random leaves). This tree fit the data significantly worse than our ML tree, or any of our candidate trees. Thus, we were able to identify a single tree with the best fit to the data (Figure 4A), and trees generated according to the null hypothesis that there was no hierarchical structure in the connectivity fit the data significantly worse.

The ML tree shows us which single tree fit the data best, however, it does not tell us how well each of the individual clusters in the tree were supported by the data. To estimate this, we fit a consensus tree to the 50 trees with the highest likelihood, from our set of candidate trees. The consensus tree contains the clusters which occur most commonly among the 50 trees we used as input and also tells us how often those clusters occurred. Thus, the consensus tree provides insight into how robust the clusters were in the ML tree. The more often a particular cluster occurs in the 50 best trees, the better supported that cluster is by the data. It is also possible for the consensus tree to be very different from the ML tree, in which case the entire ML tree would be poorly supported by the data.

We found that the consensus tree (Figure 4B) was highly similar to the ML tree (Figure 4A), and most of the clusters in the ML tree occurred often in the consensus tree. In fact, all but one of the major clusters were identical, showing that the trees that fit the data well shared structure with the ML tree. The exception was areas 10v and 10d, which clustered with dlPFC in the consensus tree, but with vmPFC in the ML tree. The cluster that occurred in the ML tree, however, occurred almost as often as the cluster which was found in the consensus tree (10v/10d/32; 15 times). Thus, it was almost as common as the most common cluster, which put area 32 by itself, and clustered 10v/d with dlPFC 17 times. Interestingly, areas 10v/d are also at the spatial border between the two groups to which they commonly cluster, and they may represent an interface between these two clusters. Additionally, although the cluster defining areas 12l, 45 and 46v is attached directly to the cluster of the insula, 12o and 13 in the ML tree, and attached above the other groups in the consensus tree, the ML algorithm assumes the trees are unrooted, so these two architectures are equivalent with respect to the likelihood values, i.e. these trees have the same fit to the data. Also, the particular cluster containing all of these groups, as present in the ML tree, occurred 9 times in the best 50 trees. Additionally, we quantitatively compared the ML and consensus trees, by carrying out our bootstrap analysis, and comparing the difference in the distribution of the likelihoods for the ML tree and the consensus tree. We found that these distributions were statistically indistinguishable (p>0.05, K-S test). Thus, the ML tree not only fits the data best, but its clusters are also common in the best 50 trees. This also suggests that, if there is a tree that fits the data better than the ML tree, its structure would likely not differ much from the ML tree.

### Tree Fit to Binary Data

**Figure 5:**
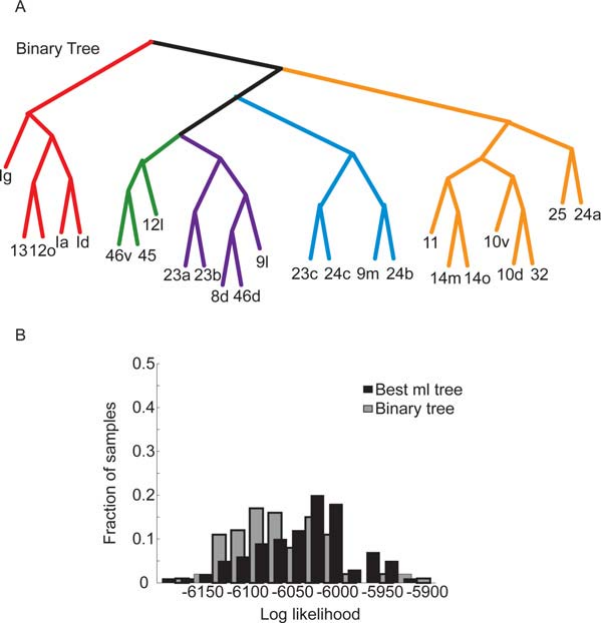
Fit of binary tree. (A) Tree which best fit the binary data. (B) Comparison of fit of binary tree and tree fit to weighted data, on the weighted dataset.

While the matrix of weighted connectivity data contains the most information, it is always difficult to accurately quantify anatomical connectivity data. Therefore, it is also interesting to examine clustering of areas based upon present/absent connections. To carry out this analysis we first converted all the data in the matrix to 0's and 1's by thresholding connections which were stronger than week (>33) to be a 1, and everything else (0–33) a zero. We then subjected the data to the same clustering analysis used to generate the tree fit to the weighted data. We identified the tree which fit the binary data best (Figure 5A). This tree was highly similar to the tree fit to the weighted data. In fact, the main clusters identified in the tree fit to the full data were all the same, although they were organized differently at higher levels of the tree. To see whether or not this tree differed statistically from the tree fit to the weighted data, we compared the fit of the binary tree to the fit of the weighted tree on the weighted data. We found that although the fits were similar (Figure 5B), they were significantly different (K-S test, p<0.01). Thus, major clusters in the data were the same whether we analyzed the weighted data or the binary data, but the organization of these clusters at higher levels resulted in significantly different fits to the weighted data.

### Dominant Inputs to Each Cluster

The hierarchical cluster analysis does not suggest that there are a specific number of clusters in the data. In fact, the hierarchical structure does not define a specific number of clusters, but rather a hierarchical relationship among sets of areas. Defining the number of clusters in a dataset is a very difficult problem, and it is not clear that it is meaningful in the situation we are studying here. We can, however, examine the dominant inputs to hierarchically related sets of areas, to gain insight into which variables particular sets of areas are processing. Thus, we have divided the major clusters in the ML tree into 5 groups, which we have labeled coPFC (caudal orbital), vlPFC (ventral-lateral), dlPFC (dorsal-lateral), dmPFC (dorsal-medial) and vmPFC (ventral-medial). These groups were found in both the ML tree and the binary tree. Interestingly, these areas correspond to mostly anatomically contiguous areas, except areas 23a/b which cluster with dlPFC, although they cluster as their own group within this cluster. Using these clusters we calculated the inputs from sets of afferent areas grouped together by functional significance. Ideally, this analysis would be based upon the total number of neurons from each group of afferent areas that projected into a cluster. However, this information is not consistently and precisely available. Therefore, we carried out the analysis using the sum, the max and the average across the inputs from each set of afferent areas. Results for the sum and the max were very similar, so only the results for the sum and the average are shown.

**Figure 6:**
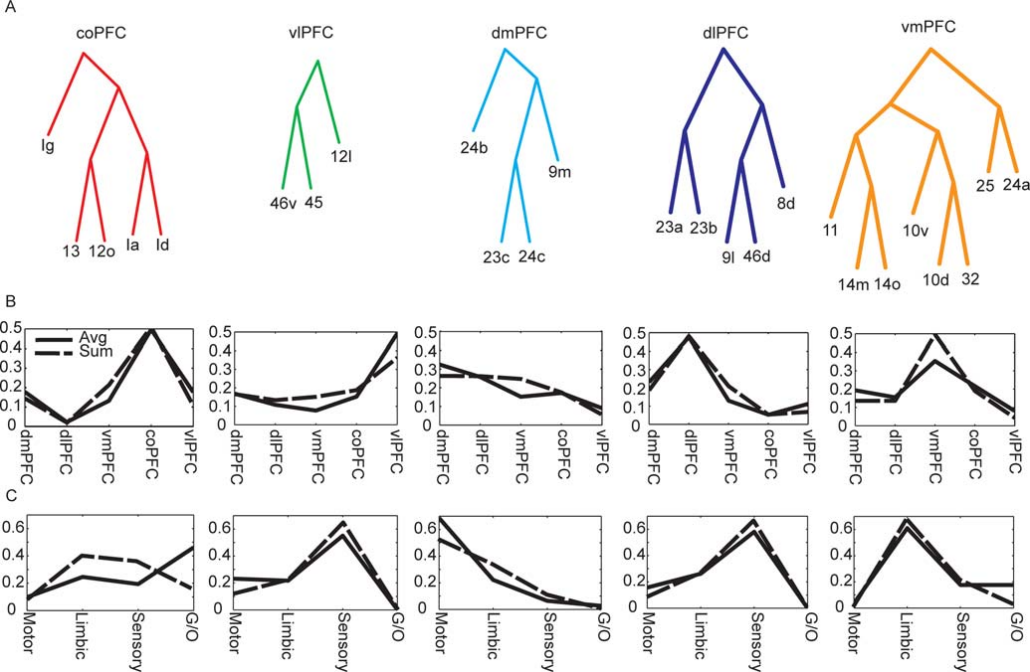
Profile of inputs characterizing each cluster of areas. (A) Clusters. (B) Intrinsic, frontal inputs. (C) Extrinsic inputs. All inputs were normalized to sum to 1. Thus, the line indicates the proportion of inputs coming from each modality.

Using the average, we found that the inputs to each cluster from other frontal areas were dominated by the other areas within the cluster (Figure 6B). This is consistent with the fact that connectivity tends to be local as discussed above, and each cluster is composed of a local group. The extrinsic inputs were also unique to each cluster (Figure 6C), as is necessarily the case because the algorithm separated the areas into clusters based upon their inputs. Generally, coPFC was defined by chemosensory (gustatory/olfactory) and interoceptive inputs, although it receives some inputs from each of the groups, as indicated by its relatively less peaked distribution of inputs. The vlPFC and dlPFC were both defined by extero-sensory inputs (visual/somatosensory/auditory and multisensory). The sensory inputs to the vlPFC and dlPFC, however, tend to originate in different parts of the posterior brain. Specifically, the dlPFC receives inputs from dorsal visual and auditory areas [15]–[17], and the vlPFC receive inputs from ventral visual and auditory areas [15],[16],[18]. Physiologically, however, these signals appear to be integrated at the level of the single neuron [19]. The dmPFC was defined by its motor input, and vmPFC was defined by its limbic inputs (hippocampus and amygdala). Thus, each cluster of areas had a unique anatomical fingerprint.

When the analysis was based upon the sum (Figure 6B and 6C, dashed lines) the results were generally similar to the results obtained with the average, except in the case of the extrinsic inputs to the coPFC and the intrinsic inputs to the dmPFC. When the sum of the inputs was used, the coPFC had more limbic and sensory inputs; in general the coPFC always had the most diverse set of inputs of any of the clusters. Although the strongest inputs to the dmPFC were still from dmPFC, this effect was smaller, and inputs from dlPFC and vmPFC, both of which border dmPFC were also strong.

**Figure 7:**
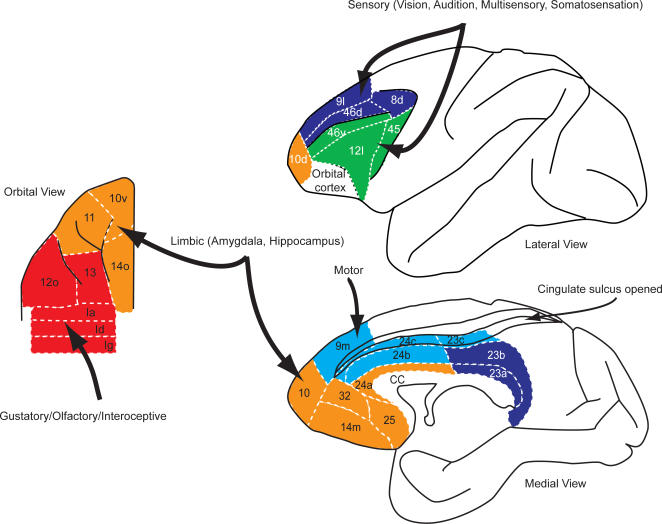
Summary of clusters of prefrontal areas and their dominant inputs.

Of course there is more detail in the connectivity of the areas than what is represented in the clusters we chose as illustrative. We could consider the distribution of inputs at finer levels of the hierarchy. However, the clusters we chose do represent a statistically robust characterization of the inputs, at a particular level of clustering, and therefore it represents a justified simplification. This connectivity profile is summarized in Figure 7 where it can be seen that the motor inputs to the dmPFC and the chemosensory inputs to the coPFC areas follow the local connectivity rule because the main inputs to these areas are from adjacent areas.

## Discussion

We carried out statistical analyses of the connectivity data for a set of 25 prefrontal cortical areas. Our analyses identified several salient features of the organization of connectivity. First, we found that signals could propagate throughout the frontal cortex in just 2 steps. This was due to the fact that each frontal cortical area sent projections to almost 9 other areas, on average. Furthermore, connections tended to be predominantly local, with areas connected to 94% of their spatial neighbors. Thus, there tends to be a lot of local connectivity in frontal cortex.

The main goal of our study was to identify the dominant connections to the frontal cortical areas, in an effort to better understand the function of each area. Ultimately, the computations which can be carried out by each area are limited by the inputs to the area, and as such, understanding the inputs can give insight into what the computations might be. We carried out maximum likelihood hierarchical cluster analysis of the afferent inputs of the frontal cortex, and found trees which provided a concise description of the organization of the frontal cortical areas. This analysis identified 5 groups of areas, each of which was anatomically contiguous, and each of which had a unique set of inputs. Because sets of clustered areas were anatomically contiguous, we treated them each as a single cluster for the analyses which examined the dominant inputs.

We also found that trees fit to binary data were similar, although statistically distinguishable, from trees fit to weighted data. Specifically, the membership at the level of clusters was similar, whereas the arrangement of these clusters was different. At a formal level, this occurs because the relative distances between areas are the same within the clusters when computed with weighted or binary data, whereas they are different between clusters. This likely comes about because of the diverse signals being processed in prefrontal cortex, and the large number of areas (25) we were considering. As shown in the results, each cluster of areas is defined by a dominant set of inputs (i.e. strong inputs). The dominant inputs to each cluster come from different modalities. For example, while dmPFC receives a strong motor input, there is very little motor input to vmPFC or coPFC. Because of this, converting the weighted data to binary data does not have a large influence in the cluster membership. Furthermore, the fact that the cluster analysis is similar for binary and weighted data, suggests that the cluster membership is driven by these strong connections. The weak connections, on the other hand, are less important for defining the clusters, but perhaps more important for defining the organization at higher levels. While this holds in prefrontal cortex, it will not necessarily hold in other systems, where graded connectivity is more relevant.

### Functional Considerations

Given that clusters of areas are defined by a dominant set of anatomical inputs, it is interesting to try to examine how these inputs can further our understanding of the function of each area. Broadly, we can consider a gradient of function starting from coPFC and vmPFC to the more differentiated areas, dlPFC and vlPFC. Evidence from several approaches has implicated the orbital and medial areas in decision making when emotion, primary rewards or conditioned reinforcers, all of which have affective significance, are involved [20]–[28]. Given our analyses, this is likely due to the representation within these areas of primary rewards and drives, brought in via the anatomical projections which bring chemosensory and interoceptive information into the coPFC, and the emotional aspects of stimuli, via the limbic projections to the vmPFC. The limbic inputs may represent learned associations between primary rewards and sensory stimuli on a longer time-scale [29], perhaps even innate associations. Lesions in either structure would lead to deficits in decision making because the affective information would not be available to the rest of the frontal network, similar to the effect of destroying early visual cortex on visual perception.

The role of the coPFC and the vmPFC in decision making motivated by affect is also consistent with the fact that the vmPFC provides the primary cortical input, along with some contribution from the coPFC, to the hypothalamus, a structure with obvious importance in emotions [30],[31]. The vmPFC and the coPFC also provide significant input to neuromodulatory systems including projections to the cholinergic nucleus which projects back to the cortex (CH4) [32],[33] as well as the locus coeruleus which provides the norepinephrine input to the cortex [34]. The vmPFC and coPFC do not, however, appear to project to the dopamine neurons in the macaque [35], and whether or not they project to the serotonin neurons in the raphé nuclei is not well known [36]. Thus, two of the neuromodulatory systems which are important in decision making derive their cortical inputs from the coPFC and the vmPFC.

In contrast to the co/vmPFC the vlPFC and the dlPFC likely play the dominant role in integrating the affective information generated in the co/vmPFC networks with rational sensory information generated in parietal and temporal sensory networks [37], which are the main inputs to the vl/dlPFC from outside the frontal networks. The rational information is important when decisions have to be based upon the statistics of sensory stimuli, where affective information plays a limited role [38]–[40]. This would be important, for example, when one was trying to resolve a road sign in a snow storm using the noisy visual input. The result of the integration of affective and rational information in lateral frontal cortex is the generation of a distribution over possible actions [41], with each action weighted by its value or one's belief that it is correct, incorporating both the rational and the emotional information. Thus, lateral frontal cortex carries out inference across multiple information sources, for action.

Single cell neurophysiology and fMRI have implicated the dmPFC in various aspects of reward guided action selection [42]–[46] and action conflict monitoring [47],[48]. This is consistent with our data showing that the dmPFC, like other frontal areas, has strong connection with its neighbors, which are the vmPFC and motor areas. The dmPFC areas are also interesting in that they have direct connections to the spinal cord and sub-cortical oculomotor areas [49],[50] giving them direct control over action. Thus, one would expect a combination of motor and affective responses. However, modulation of motor responses by expected reward have been found throughout the brain [51]–[57], making the unique role of the dmPFC unclear. Recently, interesting lesion data has suggested a role of the anterior cingulate portion of the dmPFC in guiding actions based upon the history of reinforcement [46], as well as mediating action avoidance based upon fearful stimuli [28], which is generally consistent with the physiology. Although it seems clear that the dmPFC plays some sort of role in integrating action and reward, the specific role is not yet clear.

### Conclusion

We found a statistically significant hierarchical organization of clusters in the anatomical inputs to frontal cortex. Based upon this organization, we examined the dominant inputs to each of 5 clusters. We found that the inputs to the areas within each cluster, from other frontal areas, tended to be mostly from other areas within the cluster. Furthermore, we found that each cluster had a distinctive set of inputs from outside the frontal network. Specifically, vlPFC and dlPFC were dominated by sensory inputs, although from temporal and parietal cortex, respectively. The vmPFC was dominated by limbic inputs, and dmPFC was dominated by motor inputs. The inputs to coPFC tended to be the most heterogeneous, but considering the average strength of inputs from other areas, coPFC had a strong gustatory and olfactory input. Thus, we have used the cluster analysis to define statistically robust groups of areas in prefrontal cortex, and we have shown that each set of areas has a dominant set of anatomical inputs, which likely drives the functional role of that area.

## Methods

### Compilation of Connectivity Database

The matrix of connections was compiled primarily through a direct search of the primary literature on anatomical tract-tracing studies (see Text S1) and consultation of the CoCoMac database. We focused on data using modern methods, most of which has been published since 1980. Injections were only used to define connectivity if they remained entirely within a single cyto-architecturally defined area. Most of the data comes from retrograde tracers injected into frontal cortical areas. Whenever possible, the data in the matrix was coded with respect to the strength of the connection, as this was often available. Different studies divide the connections using more or less precision. We have attempted to retain the amount of precision reported in the original manuscript wherever possible, and recoded the information into a scale of 0–100. Thus, connections described as absent (0), weak (33), moderate (67) or strong (100) were coded accordingly. In some cases in which specific strengths were not given, we examined the published figures. In our analyses we compare the results using both weighted connections, and connections coded as only present or absent. Our main conclusions are consistent with either perspective. Often a particular connection was reported in several studies or with multiple injections. Broadly speaking, most studies were in agreement. We also focused on inputs to, as opposed to outputs of, the frontal cortical areas, as these have been studied much more extensively. In many cases in which it has been examined connections are bi-directional. However, this is not always true.

The architectonic subdivisions we used were based mostly on the map used by Barbas and Pandya [58], although we distinguished between a lateral and orbital 12, as well as a dorsal and ventral 10, a lateral and orbital 14, and a ventral and dorsal 46 as was done in some studies [59]. Thus, the parcellation scheme we have used is somewhat gross, but it allowed us to integrate data across studies consistently.

### Fitting of Tree Models

We fit maximum likelihood (ML) trees [9],[10],[12],[13] to the distances between the afferents to each frontal area we considered. These trees are based upon a branching diffusion process, and they model the distance between a pair of nodes using a factored multivariate Gaussian distribution, in which the variances are used as estimates of the distances. The distances used were the sum of the squared differences in inputs, calculated as:
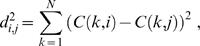
(1)where *N* is the total number of input areas we considered (68 which had non-zero inputs to at least one of the areas), and *C(i,j)* is the projection from area *i* to area *j*, (i.e. neurons in area *i* send axons to area *j*). We considered the structure of 25 frontal areas, so we calculated a 25×25 symmetric distance matrix, with zeros along the diagonal.

The ML trees allow us to do two things. First, estimate the length of individual branches, which, for the present purposes were of secondary interest, and second, estimate the likelihood of the data, given the tree structure and the branch lengths. We maximized the likelihood of the data, for a given tree *T*, by changing the edge lengths *l*. Calculation of the likelihood was done using the “pruning” algorithm of Felsenstein [9],[10], and optimization of the edge lengths is carried out with a combination of pruning and ML estimation [9], which is formally an expectation maximization (EM) algorithm. For details of the procedure, readers are referred to the original papers. We provide a sketch of the algorithm here. The PHYLIP package also contains an implementation of this algorithm.

The pruning algorithm proceeds as follows. First, a pair of nodes (*l_1_* and *l_2_*) can be pruned by replacing the distance of the branch to the pair's parent (*l_p_*) by the following:
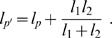
(2)The distance of the new primed node, *p*′, to the remaining leaves and other primed interior nodes can then be calculated as
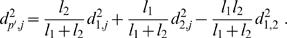
(3)This process can be repeated recursively, until the three branch lengths of any interior node (i.e. parent and two children) of the tree have been replaced by their primed lengths, by propagating lengths in from the periphery. After this, one can estimate new lengths for each of the primed branches of the interior node, using:
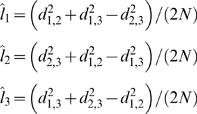
(4)Equation 2 can then be used to re-estimate the actual interior branch lengths. One then moves to a new node, and repeats this process. After a few iterations through the tree, the estimates have converged, and one can then calculate the likelihood of the tree. The likelihood is calculated on the pruned nodes. Specifically, after every pair of nodes is pruned, the likelihood of the node is given by
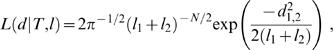
(5)where primed values are used if it is an interior node. All of the pruned nodes are independent, so the likelihood across pruned nodes can be multiplied, to give the likelihood of the tree.

Once we found the edge lengths *l* which maximized the likelihood for a given tree structure *T*, we had the maximum likelihood estimate for the tree structure, subject to possible problems with ending up in local minima. Initial values for the lengths were taken from the agglomerative clustering algorithm. Thus, they may have been close enough to the global ML value to avoid local minima. The final likelihood was an estimate of how well the tree structure, *T*, fit the data. In least-squares or linear regression, for comparison, the likelihood is a function of the unexplained variance. Thus, by comparing the likelihood of different trees, we could see which tree structures better fit the data.

**Figure 8:**
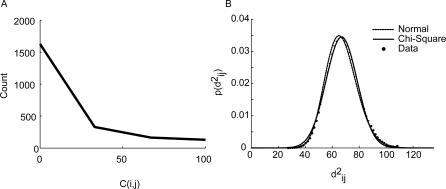
Distribution of connections and distances. (A) Distribution of connection strengths in our dataset. (B) Distribution of distances.

The connections we analyzed, *C_ij_*, took on a discrete set of values between 0 and 100, as noted above. The distribution of connections was approximately exponential (Figure 8A), which raises the question of whether or not the Gaussian likelihood function we used was valid. First, it is important to point out that sums of uncorrelated random variables, independent of their individual distributions, tend to a Gaussian distribution, per the central limit theorem [60]. This is the basis of many classical statistical analyses, including the chi-square analyses of contingency tables. Thus, because we were considering sums of distances measured across many inputs, the true likelihood function would tend to a Gaussian if we were considering an infinite number of inputs areas [61]. However, we also examined whether or not our Gaussian assumption was reasonable within our finite dataset, given that we were only considering 68 input areas. By assumption of our model, the distances given in Equation 1, divided by the variance, should follow a Chi-square distribution, with 68 degrees of freedom, as this is the distribution of the sum of the square of a standard normal random variable. We examined the distribution of 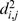 values in our dataset by first bootstrap sampling the *k* dimension, as we also did when finding candidate trees (see below) 1000 times, and computing 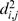 for each bootstrap sampled dataset. We then normalized these distributions for each connection *i,j*, and computed the average distribution. It can be seen that the data distribution is well fit by both a chi-square distribution (p>0.05; Kolmogorov-Smirnov test), and the corresponding Gaussian distribution, to which the chi-square distribution also tends (Figure 8B). Thus, our distribution assumption is well satisfied by our data.

Although the ML procedure gave us a tool for maximizing the likelihood of a given tree structure, it did not tell us how to find the optimal tree structure. In fact, with 25 leaves there are 4,861,946,401,452 different binary trees and 46,026,618 different tree architectures if we ignore the assignment of areas to leaves. Thus, we cannot search through the complete space of possible trees and various heuristics have to be adopted. Our procedure was as follows. First, we generated 1000 bootstrap datasets from our original dataset, by sampling with replacement from dimension *k* in Equation 1. Thus, each bootstrap dataset included a resampled set of the inputs to each area. We then fit a tree to each of these 1000 datasets, using the average linkage agglomerative clustering algorithm in Matlab. We also added the tree generated with the agglomerative clustering algorithm on the original, unsampled dataset, to the 1000 bootstrap trees, resulting in a set of 1001 trees. In pilot analyses we found the average linkage algorithm gave reasonably robust and meaningful results, and here we were only depending on it to generate a good set of candidate trees. Interestingly, the tree fit to the un-sampled dataset was ranked 49^th^ in terms of likelihood when compared to the 1000 trees generated by the bootstrap analysis and thus the standard agglomerative algorithm did not find the best tree on the full dataset. We then searched through the 1001 trees to see if there were any duplicates and found no duplicate trees. We also used jack-knife resampling, in which we built 68 distance matrices by excluding one of the inputs for each matrix. When we searched through the jack-knife trees we found only 29 unique trees, with the rest of the trees being duplicates. Because we wanted to start with as rich a set of candidate trees as possible, we used the bootstrap trees and not the jackknife trees.

To test the fit of each tree generated with our bootstrap analysis we carried out a second stage bootstrap analysis. To do this we fit each tree (*T*) generated in step 1 to 100 new bootstrap datasets, and maximized the likelihood for each tree by adjusting the edge lengths (*l*). This gave us a distribution of 100 likelihood values for each of our 1001 bootstrap trees. We were then able to compare the 1001 trees. As a null hypothesis, we also generated trees by scrambling the leaves for a particular tree architecture, and computing the likelihood in bootstrap samples.

We also fit consensus trees to subsets of the 1001 bootstrap sampled trees. Consensus trees were fit with the Consense program, which is part of the PHYLIP program for phylogenetic inference. Consense searches through a set of trees and constructs one tree which contains the most common clusters across the set. We have used this program in the past to find consensus trees from a set of trees fit to neural data [62].

Other clustering approaches to analyzing similar data have been put forward [6]. Each approach is, however, appropriate for different questions, and ours is most suited to answer the questions we have set forth. Specifically, we were interested in clustering defined in terms of inputs from outside the 25 areas we are clustering, whereas previous approaches worked more specifically with clustering based only upon connectivity within sets of areas. Additionally, our approach allows us to do hypothesis testing, and we have shown (Figure 8) that the distributional assumptions of our model are met. Furthermore, our approach relies less on the specific algorithm used to generate the trees, as we used an agglomerative algorithm to generate a set of candidate trees, but then found the best tree using the ML algorithm. We also show the distribution over trees predicted by our dataset, using the consensus analysis, which gives important information on how well the specific tree that we show as the ML tree is supported by the data. Thus, in many cases, and indeed in our case, there may be many possible trees that are all well supported by the data (i.e. have similar likelihood). Our analysis also shows individually which clusters are best supported by the data, and how well they are supported. Finally, previous authors have also used multi-dimensional scaling (MDS) approaches on similar datasets [4]. These approaches may allow one to interpret the dimensions into which the variables are projected. For example, one may find that areas can be located on sensori-motor axes, where primary sensory and motor areas lie at one end of the axes, and classically defined association areas lie at another end. However, MDS forces one to select a number of dimensions, usually 2, into which the data is projected. This is a strong assumption, and it is difficult to check in practice. Furthermore, visualization, which is a strength of 2-D MDS, is difficult in 3-dimensions [63], and impossible beyond that. The hierarchical clustering approach, on the other hand, does not require one to assume a number of dimensions, or a number of clusters. In practice, however, one may obtain comparable results with MDS analyses. Specifically, one may find that variables that are near each other in the MDS analysis form clusters in the hierarchical cluster analysis. We prefer our approach, however, as we were interested in finding clusters of areas, and the Gaussian likelihood function allows us to do hypothesis testing, as discussed above.

## Supporting Information

Text S1 Supporting Information.
(1.93 MB DOC)
