# Supplementary material for: The Statistical Neuroanatomy of Frontal Networks in the Macaque
Source: PLoS Comput Biol. 2008 Apr 4;4(4):e1000050. doi: 10.1371/journal.pcbi.1000050 (PMC2268011; doi:10.1371/journal.pcbi.1000050)

**Supplemental material:**

List of papers used to generate connectivity matrix

**Motor**

Barbas and Pandya, JCN, 256:211, 1987.

Cipolloni and Pandya, JCN, 403:431, 1999.

Leichnetz, JCN, 254:460, 1986.

Lu et al., JCN, 341:375, 1994.

Matelli et al., JCN, 251:281, 1986.

Muakkassa and Strick, Brain Res, 177:176, 1979.

Preuss and Goldman-Rakic, JCN, 282:293, 1989

**Motor/Anterior Motor Cingulate**

Bates and Goldman-Rakic, JCN, 336:211, 1993.

Morecraft and Van Hoesen JCN, 322:471, 1992.

Morecraft and Van Hoesen JCN, 337:669, 1993.

Morecraft and Van Hoesen, Brain Res Bull, 45:209, 1998.

Vogt and Pandya, JCN, 262:271, 1987.

Wang et al., Neurosci Res, 39:39, 2001.

**Cingulate**

Kobayashi and Amaral, JCN, 466:48, 2003

Morris et al., Eur J Neurosci, 11:2506, 1999.

Parvizi et al., PNAS, 103:1563, 2006

Vogt and Pandya, JCN, 262:271, 1987

Wang et al., Neuroreport, 15:1559, 2004.

**Hippocampal/parahippocampal/perirhinal/TF/TH**

Barbas and Blatt, Hippocampus, 5:511, 1995.

Insausti et al., JCN, 2654:396, 1987.

Kondo et al., JCN, 493:479, 2005.

Lavenex et al., JCN, 447:394, 2002.

Lavenex et al., JCN, 472:371, 2004.

Suzuki and Amaral, JCN, 350:497, 1994.

Suzuki and Amaral, J Neurosci, 14:1856, 1994.

**Amygdala**

Amaral and Price, JCN, 230:465, 1984

Barbas and Olmos, JCN, 300:549, 1990.

Carmichael and Price, JCN, 363:615, 1995.

Ghashghaei and Barbas, Neurosci, 115:1261, 2002.

McDonald, Prog Neurobio, 55: 257, 1998.

Pitkanen and Amaral, JCN 398:431, 1998.

Price and Amaral, J Neurosci, 1:1242, 1981.

Stefanacci et al., JCN, 375:552, 1996.

Stefanacci and Amaral, JCN, 421:52, 2000

Stefanacci and Amaral, JCN, 451:301, 2002.

**Orbitofrontal**

Barbas et al., JCN, 410:343, 1999.

Baylis et al., Neurosci, 64:801, 1995.

Carmichael and Price, JCN, 363:615, 1995.

Carmichael and Price, JCN, 363:642, 1995.

Carmichael and Price, JCN, 371:179, 1996.

**Visual/Somatosensory/Auditory**

Baizer et al., J Neurosci, 11:168, 1991.

Barbas, JCN, 276:313, 1988.

Carmichael and Price JCN, 363:642, 1995.

Hacket et al., JCN, 394:475, 1998.

Petrides and Pandya, JCN, 228:105, 1984.

Romanski et al., Nat Neuro, 2:1131, 1999

Romanski et al. JCN, 403:141, 1999.

Webster et al., Cer Cor, 4:470, 1994.

**Insula**

Mesulam and Mufson, JCN, 212:23, 1982.

Mesulam and Mufson, JCN, 212:38, 1982.

**Olfactory and Gustatory**

Carmichael et al., JCN, 346:403, 1994.

Pritchard et al., JCN, 244:213, 1986.

Pritchard et al., Exp Neurol, 165:101, 2000.

**Within PFC**

Barbas and Pandya, JCN 286:353, 1989.

*Other connections – thalamus and striatum*

Although it could be argued that we should have considered more connections in defining our clusters, many of the other possible connections show patterns which are consistent with our clusters. Two subcortical targets, the thalamus and the striatum are especially conspicuous, although connections with the striatum would be outputs, and not inputs, they are obviously patterned and important. However, projections to both these structures are broadly organized in a way that is consistent with our findings. For example, projections of frontal areas to the thalamus, most of which go to the medio-dorsal nucleus (MD), are consistent with our clusters (Giguere and Goldman-Rakic, 1988; Barbas et al., 1991; Siwek and Pandya, 1991; Ray and Price, 1993), and a similar consistency has been shown for projections to the striatum (Kunishio and Haber, 1994; Ferry et al., 2000; Haber et al., 2006). Specifically, the phylogenetically older areas vmPFC and coPFC project ventrally into the striatum, and as one moves away from these structures towards more differentiated areas through either vlPFC or dmPFC and dlPFC, one moves dorsally in the striatum. Correspondingly, the vmPFC and the coPFC project medially in the MD nucleus as well as to midline thalamic nuclei, and as one moves away from these areas, projections go to more lateral areas of the MD nucleus. Thus, including these subcortical targets in our analysis would have had a minimal impact on the clusters to which each area was attributed.

References:

Barbas H, Henion TH, Dermon CR (1991) Diverse thalamic projections to the prefrontal cortex in the rhesus monkey. J Comp Neurol 313:65-94.

Ferry AT, Ongur D, An X, Price JL (2000) Prefrontal cortical projections to the striatum in macaque monkeys: evidence for an organization related to prefrontal networks. J Comp Neurol 425:447-470.

Giguere M, Goldman-Rakic PS (1988) Mediodorsal nucleus: areal, laminar, and tangential distribution of afferents and efferents in the frontal lobe of rhesus monkeys. J Comp Neurol 277:195-213.

Haber SN, Kim KS, Mailly P, Calzavara R (2006) Reward-related cortical inputs define a large striatal region in primates that interface with associative cortical connections, providing a substrate for incentive-based learning. J Neurosci 26:8368-8376.

Kunishio K, Haber SN (1994) Primate cingulostriatal projection: limbic striatal versus sensorimotor striatal input. J Comp Neurol 350:337-356.

Ray JP, Price JL (1993) The organization of projections from the mediodorsal nucleus of the thalamus to orbital and medial prefrontal cortex in macaque monkeys. J Comp Neurol 337:1-31.

Siwek DF, Pandya DN (1991) Prefrontal projections to the mediodorsal nucleus of the thalamus in the rhesus monkey. J Comp Neurol 312:509-524.

Table showing raw connectivity data which was analyzed.


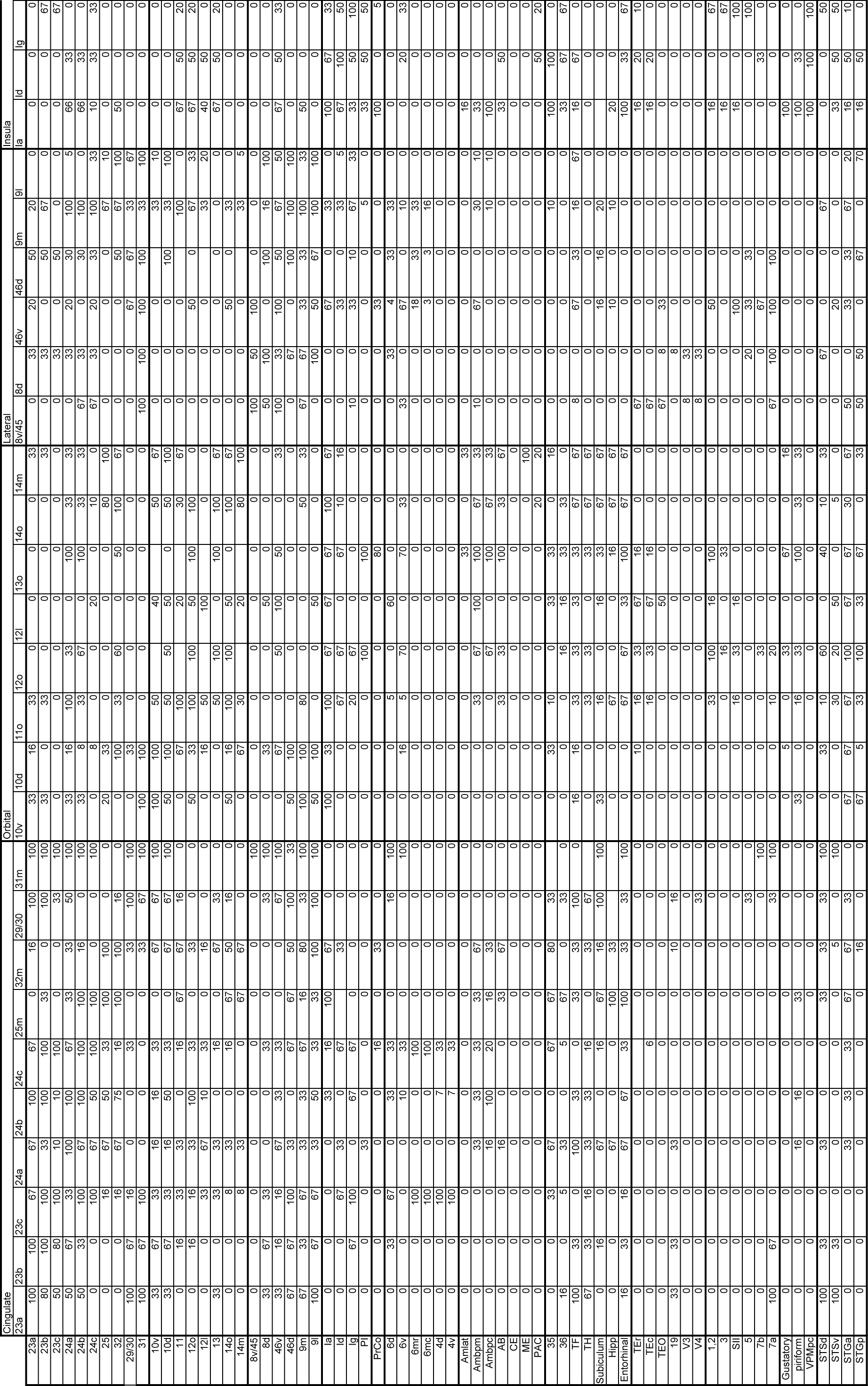

Supplement: Text S1 — Supporting Information. (1.93 MB DOC) [file pcbi.1000050.s001.doc]
